# Short History of Epidemiology for Noninfectious Diseases in Japan. Part 3: Nutritional Dystrophia

**DOI:** 10.2188/jea.18.45

**Published:** 2008-04-10

**Authors:** Kunio Aoki

**Affiliations:** 1 Professor Emeritus, Nagoya University, and Honorary President, Aichi Cancer Center.; 2 Chairman, The Nagoya City Council of Social Welfare.

## INTRODUCTION

The reality of defeat in the war and surrender on August 15, 1945 came as an extraordinary shock. On the contrary side, for the people of Japan the war’s end was more in the nature of a relief from grief for the many victims of the long war, from fear of air raids, from suffering due to wartime shortages, from severe restrictions on daily lives, and, in particular, freedom from blatant spiritual and psychological pressures from the Japanese military. However, as far as people’s lives were concerned, in addition to food shortages, war’s end brought loss of many workplaces and private properties. As a result, nutrition disorder had to be endured by the majority of people. In some, nutrition disorder progressed to dystrophia. Many died without receiving nursing or care. The author himself experienced the misery of the time’s undernourishment. He also eye witnessed several patients suffering from malnutrition or various types of nutrition disorder. Today, half a century later, the experience of such miseries and hardships appears to have been forgotten. Given that these took place immediately after the end of the war, literature on dystrophia is extremely scarce. Complete documents are meager. As a result, we live in a time when even professional researches do not know, for instance, how many people actually died of malnutrition. Viewing works on the history of epidemiologic studies on non-infectious diseases in Japan, the author considers the topic of dystrophia to be an important one that should not be overlooked by scholars. While the author is not a nutritionist and may not be competent enough to take up the challenge, he has ventured to collect records of the time to gain an epidemiologic overview. Now in this time of nutritional excess, nutrition disorder has once again become a major problem. Clarifying the characteristics of disorders of undernourishment may be useful as well in dealing with nutritional excess. As the materials the author could collect are quite limited in scope, this epidemiologic review is an incomplete one. Nevertheless, the author believes the work may in the future serve as a reference.

## WARTIME INCREASES IN MALNUTRITION DISORDERS AND THE RESEARCH COMMITTEE

Dystrophia is a well known name for a disease that has frequently appeared in defeated countries, such as Germany after World War I. For some of the people who suffer malnutrition caused by food shortages, dystrophia can be a fatal disease which, combined with other external or internal factors, exhibits as a specific syndrome. Further, dystrophia’s clinical conditions appear to differ superficially from person to person, or to depend on the group affected. Therefore, it appears to have been considered doubtful whether general characteristics of dystrophia could be defined and studied in the way other general diseases can.^1^

During the 1940s, reports appeared in Japan of dystrophia among soldiers dispatched to China or other foreign countries. In and after 1942 in Japan as well, observations of dystrophia were not infrequent. With increased food supplies, the majority of dystrophia cases would disappear. However, in the Japan of the 1940s wherein food was scarce, it was thought that prevention could be possible to a considerable degree. At that time, in addition to improving food procurement, research efforts began to focus on necessary dietary nutritional contents as well as improved dietary habits.

From the 1930s on, owing to Saiki’s^2^ efforts, nutritional science was recognized as a division of medicine and was beginning to be systematized. The policy imposed on “a wealthy nation with a military power” aided these moves. Centering on developmental phases, basic and practical studies continued on posture, physical constitution, athletic prowess, characteristics of nutritional elements related to resistance, composition of ingested nutritional elements, selection of foods and how to cook, and dietary habits. Many studies on vitamin deficiency were among the disease-related studies. Under the wartime food restrictions, researchers in the field of hygiene began to study nutrition, cooking, menus, and ways to adapt people’s lifestyles to these conditions; further, research discussions continued on acclimatization of nutrition in tropical or cold regions outside Japan. These studies document the urgency of food and nutrition problems in the course of the war.^3^^-^^5^

Following 1940, as needs for military provisions expanded, the quantity of food allotted to the general public decreased. As the years went by, the effects of a reduced food allotment increased. From around 1941 on, the number of undernourished patients began to rise. By 1943, records in general hospitals of likely dystrophia patients would emerge. In the background of this phenomenon were generally reduced food supplies to the public as well as distribution delays of foods, such as rice. After the end of war, the quality and quantity of rationed food further worsened. Severe drought and continual rain that began in September of 1945 contributed to the drastic drop in rice harvest, 68.8% lower compared to the previous year (anticipated yield was 5,870,000 tons), the worst harvest through the years of Taisho and Showa. The harvest of sweet potatoes, which had played the role of supplementing rice, was also quite poor. Accordingly, food distribution was not only meager, but also tended to be delayed from summer to autumn that year. In contrast, black markets flourished in the streets. Prices of cereals escalated. In the off-crop transitional season between the year’s end and spring, the mass media reports predicting that 10 million would die of starvation fanned people’s anxiety. As a matter of fact, the number of dystrophia patients in hospitals had surged from the end of 1945.

During the war, no research article on dystrophia with the word “epidemiology” in the title was to be found in Japan. The reason probably was that “epidemiology” had not been used for anything other than communicable diseases. “Abstracts of Nutritional Science,”^6^ published by Eiyo Kagaku Kenkyukai (Nutrition Research Group) in 1940 contained four articles from “Gun’idan Zasshi (Journal of Military Physicians)” under the section “war related dystrophia” observed in China. It is unclear to what extent domestic researchers understood the significance of these articles. At the end of 1944, the Ministry of Health and Welfare (currently Ministry of Health, Labour and Welfare) organized a research group^1^ as the Fifth Group (Dystrophia), the Ninth Division, Science Council of Japan. The need to identify the nation’s true state of nutrition must have forced this action. However, the above mentioned group never met during the war.

## DYSTROPHIA RESEARCH

After the war, the Japanese Government induced the previously organized group to meet in a rush to grasp the true state of nutrition in the country. In order to obtain the indispensable aid from the General Headquarters of the Allied Powers (abbreviate: GHQ) essential to navigate through the postwar food crisis, the true state of circumstances regarding food and miserable malnutrition was needed as evidence.

## REPORT FROM THE DYSTROPHIA RESEARCH COMMITTEE, THE SCIENCE COUNCIL OF JAPAN

The Dystrophia Research Group held their first meeting in September 1945, following the war’s end. The group was headed by Yasuzo Sakaguchi, Professor of the University of Tokyo. The following accounts by Professor Sakaguchi were found in the interim report^1^ of September 1946: The committee held meetings six times to discuss the circumstances of dystrophia incidence, cause, etiology, pathology, syndrome, prevention and therapy. Conclusions, however, had not as yet been reached. However, the Ministry of Health and Welfare, in need of a report to survey the state of national nutrition, asked the Committee to publish an interim report. Incidences of dystrophia, called starvation edema or postwar edema, had been high in post-World War I Germany and other places. During the war, Japan also experienced outbreaks of severe cases of dystrophia, called by the Army “war-related nutritional disorder,” by the Navy “unmanageable whole body emaciation,” or simply “malnutrition” in medical or civilian circles. The Science Council of Japan regarded the word dystrophia as not a properly academic medical term. However, in view of the popularity of the term dystrophia, research work would for a while continue calling the disease as it had formerly been known. Deciding regarding on making a rational name change when the essential nature of the syndrome had become clarified, the Committee for the time being settled on the term “malnutrition dystrophia.” The Committee began deliberation after deciding that the research would not include vitamin deficiency diseases, such as beriberi. The interim report evinced the puzzlement the committee must have felt in the face of the lack of an as yet clear concept. However, the report contained much discussion on previously published information on malnutrition dystrophia, results from new surveys as well as reviews on the clinical state, its course, and prognosis.

According to Professor Sakaguchi, the causes of malnutrition dystrophia were insufficient energy intake as well as deficiencies in protein quality and quantity. He stated that when specific external or internal factors contributed to this state, malnutrition dystrophia would occur, clearly distinguishing it from vitamin deficiencies. The report summarized the malnutrition dystrophia’s course describing various subjective and objective syndromes, results of patients’ serum and urine test, or changes in basic metabolism. While diagnosis was easy for severe cases, it was difficult for mild cases. When complications existed, it was difficult to decide the main cause. It was important to differentiate malnutrition dystrophia from beriberi, chronic communicable diseases, ulcer of digestive organs, hepatic cirrhosis or other diseases of internal organs. The possibility of early diagnosis on the basis of blood findings was discussed in another part. The primary measures of malnutrition dystrophia prevention were supposed to be replenishing energy and guaranteeing the quality and quantity of protein. As to measures for nutritional deficiency, the report listed the following advice: avoid exhausting one’s physical strength, pay attention to dietary balance, cooking methods, menu planning, or chew food well. Therapy for relatively mild cases included: bed rest, keeping warm, and diet therapy. For severe cases, blood transfusion would be an effective method, but the securing of blood would be a difficult task. Other therapies deemed beneficial were amino acid preparations, hormone preparations, or boiled bone marrow juice. These were guidelines and measures of agonizing contents handed down at a difficult time when foods as well as medicines were scarce. Sakaguchi gave a speech on the results of the Research Group:^7^ clinical conditions and outbreak mechanism of malnutrition dystrophia, explanations on food elements and metabolic aspects of digestion or absorption. At the same time, he proposed practical preventive measures, emphasizing the importance of prevention. While the author could not obtain the discussion records of the research group’s meetings, many postwar researches on malnutrition dystrophia have cited the group’s report. The author endeavored as much as possible to collect and review such research articles, in particular, contents of epidemiologic surveys. The following is an overview of the author’s effort.

## WEIGHT REDUCTION AND QUANTITY OF FOOD INTAKE DURING AND AFTER THE WAR

Behind malnutrition dystrophia, lurked nutritionally disadvantaged people. As described above, since 1941 the food situation had deteriorated steadily. Rationed food diminished by the terminal phase of the war. Owing to political and administrative confusions of the postwar era, war-devastated living conditions facilitated a sudden increase of malnutrition dystrophia.

In 1947, Kenta Omori promptly published a monograph titled “Eiyo Shitchosho (malnutrition dystrophia),”^8^ an integral work on the topic, citing numerous domestic and foreign literature as well as many information from the malnutrition dystrophia research group’s reports. He began with reference to the nutritionally disadvantaged of wartime. Already from around 1940 the same conditions as malnutrition dystrophia had been observed in Osaka, followed by Kyoto. The same condition was reported in Tokyo in 1943. He also mentioned the tendency to weight loss as a precursor to malnutrition dystrophia. The book introduced the “Wartime survey on trends concerning affecting the population’s physical strength,” a 1944 survey by the Health Science Section, Research Institute of the Minister of Health. This was a survey that traced changes in body weight beginning in 1939. The survey involved 596 railroad employees as well as 1,234 elementary school teachers in five cities. Their body weight began to gradually decrease every year beginning in 1939: the loss for railroad employees was 2.52kg a year, for school teachers, 1.66kg a year. Also reported was weight loss among pregnant women in Tokyo and Osaka. The weight loss among newborn babies in Osaka was approximately 50g. Another cited report authored by Shoji Kondo referred to height and weight loss among school children in Sendai. While a check on the school hygiene statistics collected by the Ministry of Education, Science and Culture (currently Ministry of Education, Culture, Sports, Sciences and Technology) between 1937 and 1945 revealed that pupils and students had already begun losing height and weight since around 1941, the trend was less steep in agricultural areas than in cities. Home surveys in metropolitan areas found energy intake began to decrease from around 1944: the trend was especially prominent with protein intake. It was pointed out that food rationing for employees differed considerably at their facilities depending on types of work. Namely, food distribution was not equal. Food supply tended to decrease since four years before the end of the war, differing according to areas or work facilities. While the proportion of persons with nutrition disorder differed among individual groups, it gradually and progressively worsened in all groups.

Between 1938 and 1946, Inoue^9^ studied statistics drawn from physical examinations of students who took entrance examination for the nursing school at Kyoto Imperial University. Average weights began to go down drastically from fiscal year (FY) 1940 followed by further reduction in FY 1945. Even in the postwar FY of 1946, students’ weight did not return to the original level. Weights of Kyoto Imperial University students (aged 21 years) similarly decreased from 1940. Similar trends were reported from many parts of the nation. A January 1946 nutrition survey by Fukuda,^10^ involving 177 nursing students, 15-20 years, of the Chiba Medical College, reported their daily energy intake was low at 1500kcal and 5g of protein. The rate of nutritional disorder was higher the longer students lived in dormitories (upper class students) away from home. Apart from new students, the rate of those with beriberi was a high 20%. Blood pressure was generally low. The above data revealed that because of food scarcity, from early on the number of the undernourished was high.

Sato^11^ surveyed Tokyo area residents as well as factory workers who lived in dormitories in 1946. The above survey found lower body-weight ratio among people who solely relied on rationed food than those who had other sources for food. The report displayed a great effect of food shortage on body weight, but also that the frequency of nutrition disorder depended on persons’ socioeconomic circumstances. In the report of a human experiment by Inoue,^9^ body weight decreased when food intake was below 1750kcal and 75g of protein per day for men, while with women the corresponding numbers were below 1550kcal and 70g of protein. Simultaneously, diminished work capacity was manifested in the form of nutrition disorder even at light work. Other than protein, the role played by the quantity of fat intake was considered important as well.

Omori^8^ reported changes in the quantity of nutrition intake in different areas: energy and protein consumption among Tokyo residents decreased since 1942. Similar trends were observed in Osaka, Nagoya, and Kyoto. He also introduced 1944 report by Masataro Arimoto; while energy intake of over 2200kcal a day at armament plants was good, there were differences among facilities. Postwar survey by the Ministry of Health and Welfare also found obvious differences in nutrition intake depending on the area of residence or socioeconomic class. That is to say, depending on the area or group, distribution of nutrition disorder was quite uneven.

Below is a list of relevant items from the “Chronology of Japanese dietary habits^12^” from Saito.

February 1940: promulgation of a food rationing system; rationed rice mixed with imported rice; limitation on sugar purchase; food price regulation owing to rising prices; special food supply for pregnant women and infants

April 1941: a rice ration passbook went into effect, 2-go and 3-shaku (330g) per person per day; regulation promoting school lunch (providing lunch to physically weak children and children from poor household); deepening shortage of vegetables. Decision by the government to compulsively purchase rice announced in the Government Control Outline. Rationing of fish, egg, and so on.

1942: Rationing of salt, Miso (fermented soybean paste), and Shoyu (soy sauce); promulgation of Food Management Law; Regulation for the Promotion of Agricultural Production. Report that purchases of 45-49% of fresh fish, vegetables, or dried foodstuff were at black market prices.

1943: Partial substitution of potatoes for rice rationing; government encouragement of strengthening food self-supply program. Strengthening oversight for procurement of food from sources outside the official channels; beginning of evacuation of school children into areas where air raids were unlikely.

1944: Mobilization of five million school children/students for food supply expansion; mobilization of students studying in university agricultural departments to be officers of a food supply expansion corps; closing high-class restaurants, bars, or liquor stores; opening of gruel restaurants; abolishing sugar rationing; decision to evacuate school children in metropolitan areas into areas where air raids were unlikely.

1945: In July, ten percent reduction of rationed rice to 2-go and 1-shaku per person per day; abolition of special rations for laborers. In August 1945, just after the war, the government issued an emergency administrative ruling for securing food. In September, the government submitted a food supply plan for the public to the GHQ, proposing 1551kcal per person per day. In September, black markets were opened in the Tokyo Metropolitan area. In October, the government applied to the GHQ for food import and obtained permission in November. A mass meeting for the measures against starvation death was held in the Hibiya Park, Tokyo. Around the time of the mass meeting, increased numbers of deaths caused by starvation in the Ueno station were reported. In December, at the behest of the GHQ, the Ministry of Health and Welfare conducted a Tokyo residents’ nutrition survey.

1946: In January, arrival of approximately 900 tons of wheat flour from Manila; In March, arrival of 25,000 tons of wheat from the United States. The number of black market shops increased to 60,000 in Tokyo. Because of short food supplies, an ordinance was issued to limit the number of people moving into cities from rural areas. In April, the average delay of rice distribution in Tokyo was 3.7 days, which worsened in June to 18.9 days. In May, there was a large scale May Day demonstration for food.

In October 1946, the Agricultural Land Reform Law was promulgated. In November, as the year’s rice harvest came to be known as an average one, the amount of rationed rice was raised to 2-go and 5-shaku. The sweet potato harvest was also abundant so that its consumption increased as well. In December, a survey by the Ministry of Finance announced that energy intake of a person per day was 1380kcal, 70% of household expense was spent on food; however, salaried employees’ energy intake increased to 1900kcal per day on average. Considerable differences began to emerge in energy intake among people.

In 1947, as rice collection from farmers lagged, the government took forcible measures to collect appropriate amount of crops. In the off-crop month of June, delays in rice distribution worsened again, reaching 13.2 days in Tokyo, 9.7 days in Nagoya, and 7.1 days in Osaka: in July, the delay was over 20 days. Increases in deaths due to starvation were reported. In October, Judge Yamaguchi of the Tokyo District Court, who refused to buy rice on the black market, died of malnutrition dystrophia. In November, with the arrival of a new crop of rice, delays in rice delivery disappeared. Despite the situation in Japan as it was, the Japanese people’s average life expectancy increased to over 50.

In March 1948, the government announcement that the delivery of rice exceeded the target, having the prospect for continuation of the rice rationing system. In August, the GHQ publicized that “daily energy consumption in Japan increased from 1300 to 1440kcal (2-go 7-shaku) a day”; the September announcements of GHQ regarding food distribution and economic stability were put into practice in November. Food conditions generally improved from around October, with the removal of control on fruits and increases in sugar imports and other measures in place. In 1949, control on vegetables was abolished and free sale on the market of sweet potatoes after fulfillment of the delivery quota was authorized. The average life expectancy exceeded 55 years.

The nation as a whole had suffered about 10 years of food shortages.

## REPORTS ON MALNUTRITION DYSTROPHIA

Omori reviewed and analyzed the relationship between nutrition intake in the military and malnutrition dystrophia, presented at the Research Committee of the Science Council of Japan. Sex, age, work contents, and season were factors that influenced malnutrition dystrophia: especially strong was the effect of chill on malnutrition dystrophia. He summarized malnutrition dystrophia’s clinical and pathological states. While variable, depending on the types of complications, he estimated the case-fatatlity rate to be 10-20% from various literatures. He also estimated its prevalence among Tokyo residents to be 5%. As to prevalence, which differed greatly depending on individuals’ areas of residence or groups they belonged to, his estimate varied widely, between 1-26%. Based on the conditions as of 1946, he estimated more than 10 million starvation deaths. but it was far off fortunately.

## MALNUTRITION DYSTROPHIA IN THE MILITARY SERVICES

As described above, there was a report^6^ on a malnutrition dystrophia outbreak in military troops dispatched to foreign countries beginning around 1940. Hiraki^13^ reported that since 1938, the Army reported many malnutrition dystrophia cases that occurred in China. He also introduced the presence of over nine reports on malnutrition dystrophia before 1947 Japan. Malnutrition dystrophia cases reported by Hiraki involved 1944 new draftees. Some newly drafted soldiers would lose a great deal of weight within 2-3 months, then, die, following symptoms of malnutrition dystrophia. The frequency of malnutrition dystrophia decreased after 2-3 months beyond entrance to military service. The problem responsible for this phenomenon was presumed to be the drastic changes of living conditions. Observing soldiers suffering from malnutrition dystrophia shipped back from abroad, Hiraki stated that the soldiers’ cases decidedly different from those in Japan, but similar to postwar edema patients observed in post-World War I Germany or other places. From Katsuyoshi Shimizu’s materials relevant to malnutrition dystrophia published in 1988, Shunichi Yamamoto presented circumstances of malnutrition dystrophia incidence among Japanese soldiers in China from 1938 on.^14^

According to the report of Tamotsu Sugita of the Japanese Navy, sailors’ body weight began to decrease in 1944 without any sign of recovery. A sudden drop occurred in December, particularly evident in training squadrons. During January to June of 1945, approximately 6,000 malnutrition dystrophia cases occurred in air squadrons or among students that showed a marked drop in body weight, of whom 500 died. Those persons’ total energy intake was between 2150kcal and 2870kcal per day, at the same time maintaining a protein intake of 80-90g. However, the effects of grueling training and related work coupled with exposure to cold were factors that were believed to have led to the high prevalence of malnutrition dystrophia in this group. The tendency of rising patient numbers continued until June, 1945, then began to decrease. This fact made Sugita to attach special importance to the role cold played.

Based on observations of a naval personnel isolated on the outer lying Palau islands, Tatsuhiko Tsuji^16^^,^^17^ reported 14.3% mortality (1,151 deaths) from malnutrition dystrophia out of 8,200 during January to September 1945. The number of deaths was particularly high in June and July, when total daily energy intake went down to 600kcal. On the other hand, the Army’s mortality was 8.8% of 21,069 stationed in Palau, despite the supply of a staple diet of 1200kcal, which, low as it was, the Army managed to maintain. The mortality above included deaths from communicable diseases. From this fact, Tsuji suggested that factors other than food affected mortality as well. He also recollected how food was secured at the time in order to fight malnutrition. He performed statistical analyses on 70 deaths from malnutrition dystrophia. As energy intake in summer went down below 1200kcal per day, the number of deaths increased. Patients were most numerous below the rank of petty officer; the majorities were younger than age 25 years. Overwork was also an additional factor. The average length of time between the onset of illness and death was a relatively short 47.63 days. In the majority of the cases, the onset of disease took place when daily energy supply was at a level of 1100-1200kcal. Kokichi Tsuchiya^18^ accounted food conditions on Yap Island that had worsened during 1944. Aiming at food self-sufficiency by planting sweet potatoes and other crops in September, malnutrition dystrophia increased with further deterioration of the food supply in June 1945. Over half of these cases were patients recovering from amebic dysentery. The prognosis of malnutrition dystrophia was unfavorable even after recovery from dysentery. While the only effective therapy was blood transfusion, blood donors were scarce. According to the detailed survey report by Jiro Miyazaki,^19^ on this solitary island (a battle field), where a reduced diet was of necessity since April 1944, weight reduction or subjective symptoms appeared approximately one month after decrease of energy intake had begun. A total energy intake of 856kcal in July was the minimum recorded. A sharp increase of malnutrition dystrophia occurred by January 1945, followed by many deaths. After food aid from the US military began with the war’s end, malnutrition dystrophia disappeared. Miyazaki also observed severe cases of mental disorder associated with worsening nutritional conditions. While these case reports showed differences with regard to clinical symptoms or the course of disease, at the terminal stage cachexia was common.

In March 1946, Sato^20^ did a nutritional survey of returning soldiers and laborers from the southern front. The body weight in the majority was lower when returned compared to before their departure - the larger was the weight loss, the lower was their work capacity.

## MALNUTRITION DYSTROPHIA AMONG CIVILIANS

With regard to civilians, Omori^8^ and Inoue^9^ presented their work on the increase of malnutrition dystrophia since 1944 among detainees at the Kyoto Detention Center. Among the prison inmates not many malnutrition dystrophia patients were recorded. The total energy in meals given to detainees at the detention center was 1561kcal with 55.2g protein per day. When a measure to further reduce energy intake for detainees was taken, malnutrition dystrophia cases increased. In contrast, the daily level of 2259kcal with 77.1g of protein was maintained for prisoners. This was the reason why the incidence of malnutrition dystrophia did not increase among prison inmates. Both reports indicated the close relationship between the amount of food intake and malnutrition dystrophia incidence. Generally speaking, more elderly persons tended to be affected by malnutrition dystrophia.

Matsunaga et al.^21^ reported 30% of the dead found on streets of postwar Tokyo were due to malnutrition dystrophia according to autopsy diagnosis.

Hagiwara^22^ recorded wartime dietary habit in detail. Food shortage intensified beginning in 1944. While malnutrition dystrophia incidence occurred when average energy intake went down below 1900kcal, the level was reached already in 1943 in Tokyo and Osaka. No data existed regarding actual conditions of malnutrition dystrophia among school children, evacuated to live in rural areas away from home and had to live under the wartime condition of severe food rationing.

## INOUE’S REPORT ON MALNUTRITION DYSTROPHIA AMONG THE JAPANESE^9^

In 1947, Inoue (Department of Internal Medicine, Kyoto University), a member of the Nutrition Dystrophia Research Group of the Science Council of Japan, energetically kept pursuing research on the disorder. He presented a paper on the assigned topic “Nutrition among the Japanese,” at the 44th Meeting of the Japanese Society of Internal Medicine and the 88th Meeting of the Japanese Society of Gastroenterology of 1947. The research, carried out at a difficult time, was extensive and was later published as a monograph. Below are summaries of the book’s contents, discussions of the literature, statistical observations, etiology, prevention, improvement of national nutrition, etc.

Inoue straightened out the concept of the so-called malnutrition dystrophia. With the support of the research committee members, he surveyed eight universities and three hospitals nationwide and checked 8,421 cases for circumstances of incidence, age and sex distributions, occupation, clinical state, the course of the malnutrition dystrophia etc., and found institutional and generational differences in diagnostic standards: patients’ clinical states also were not uniform. Due to the need of a facility where more objective research on malnutrition dystrophia could be carried out, Inoue chose the First Department of Internal Medicine at Kyoto University. From August 1942 through February 1947, every nutrition-related disease diagnosed at the out-patient clinic was analyzed, with the exception of beriberi. The total patient cases examined numbered 737 (5.7% of all the new patients.) Even before 1942, a small number of malnutrition dystrophia patients were diagnosed at the clinic. While the number in 1943 was 37, it increased to 160 in 1944 -- 5.3% of all the patients who visited the out-patient clinic. The number doubled to 323 in 1945 (10.7% of all the patients who visited the clinic.) A sharp increase occurred toward the end of 1944. Of these, the number of cases diagnosed as malnutrition dystrophia between August 1942 and February 1947 was 655 (90.2%) -- 5.7% of all the new patients who visited the clinic. Approximately 10% were patients with other diseases than malnutrition dystrophia. Below are the disorder’s clinical epidemiologic features. Patients increased in number during the cold season, men outnumbering women; age-wise, more were either young or middle aged, but the numbers of juveniles or the elderly also were not small. By occupation, few were in agriculture; many were factory workers, but office workers or persons without jobs (mostly housewives) were not negligible in number; students also were not an exception. Classifying patients into categories of mild (no therapy required), severe (little improvement with therapy), or in-between these two, only the severe group clearly manifested a common syndrome thereby making it possible to judge that the syndrome could be treated as an independent disease. Severe cases were most numerous in the 40-years and above group. The majority followed a chronic course, while some took acute forms. From the observations of these patients taken with a clinico-pathophysiologic eye, together with autopsy findings, Inoue depicted the overall picture of malnutrition dystrophia, particularly clearly depicting clinical characteristics of severe cases. From the inspection of surveys conducted in various areas since 1946, he estimated the number of latent malnutrition dystrophia cases to be considerably high. While the fatality rate of hospitalized patients was 4.2%, it was a high 31.7% at a prison according to surveys.

Inoue considered that it appropriate to understand the cause of this disease as multifactorial. As external factors, he counted food quantity and quality, overwork, sleep, and environmental temperature, and, sex, age, constitution, gastrointestinal diseases, autonomic nervous system diseases, endocrine secretion, abnormalities of diencephalons or pituitary system as internal factors. Inoue pointed out that patients’ clinical states would differ depending on the combination of these factors. He also demonstrated how food intake was connected to the reduction of energy, protein and fat. For prevention, he actively promoted food improvement, and passively proposed the same points stated in Sakaguchi’s summary. He also proposed early diagnosis as a preventive measure against cases becoming more severe.

As to the improvement of national nutrition, Inoue discussed food quantity and quality, at the same time emphasizing the problem of fat. Also mentioned were food allotment, rationing, or use efficiency. Further, he lectured the importance of systematic surveys of national nutrition.

Building on the results of the national nutrition surveys and following Inoue’s talk, in 1948 Keizo Kodama^23^ in his “Considerations on the future of national nutrition in Japan” discussed a Japanese national food policy. The large issue was how to conquer the problem of nutrition disorder in a practical way.

## POST 1946 GOVERNMENT MEASURES FOR MALNUTRITION DYSTROPHIA

As had been described by Saito,^12^ even though the government took several measures to alleviate malnutrition dystrophia problem, during wartime welfare of the public was just about ignored As the war ended, however, the government made efforts toward working out effective measures, at the same time petitioning to the GHQ. Permission for food imports had already been granted, resulting in some wheat imports in the course of February 1946. The Emergency Ordinance for Food issued in February enabled legal purchase for distribution of stored rice, wheat, or barley from reluctant farmers. According to Hagiwara,^22^ at this time, 130-koku of rice, commandeered by the Allied forces from the Japanese military at the end of war, was released by these forces to the Japanese government. This amount of rice distribution to the public served as stopgap rationing.

It was difficult for a defeated country to ask for immediate, unconditional aid in 1945, a year of worldwide crop failure. Therefore, the GHQ ordered the Japanese government to carry out a national nutrition survey that could serve as an attempt to base decision-making on objective data from said survey’s findings. The Ministry of Health and Welfare conducted the first residents’ nutrition survey in Tokyo during December 1945. The survey covered 31,968 (14,457 men and 17,511 women) residents of 35 wards of Tokyo. Early results published in December were as follows: people with below average body weight were at 55% for men, and 36% for women; the prevalence of residents with indices of malnutrition dystrophia (anemia, angular cheilitis, glossitis, edema, and menstrual disorder) was approximately 3.5-10%, approximately 3% with chronic diarrhea, and approximately 2% with bradycardia.^24^

Faced with these results and realizing the urgency of the situation, the GHQ decided in February 1946 on an emergency aid and released flour. The Tokyo Metropolitan Government surveyed the postmortem causes of unnatural deaths that occurred during April 1946. Deaths in the street accounted for 35%. The flash report on autopsied 186 cases included 30% of nutrition related deaths^25^: 11 from general weakness, 27 due to starvation, and 14 due to malnutrition dystrophia. The survey results later were reported in a medical journal.^21^ In the winter of 1946, a national research institute investigated the reality of winter life for 619 Tokyo residents with regard to residence, income, Engel’s coefficient, existence of home garden, and so on. Households that suffered war damage lived in an extremely narrow space; more than 60% of their cost of living was for food, a figure more than 10% higher than the 49% that rural families spent on food. Naturally, war damage greatly influenced postwar life. Malnourished persons’ blood and urine findings, based on data from weight, height, tendency to fatigue, proteinuria, blood pressure, malnutrition dystrophia, basic metabolism and other tests, were arranged in a list. Blood and urine abnormalities of the surveyed were also organized in published articles.^26^^-^^28^

Based on the nutrition survey of the Tokyo Metropolitan government, the Ministry of Health and Welfare planned a nationwide nutrition survey. The survey that began in May 1946 covered five major cities including Tokyo, Osaka, Nagoya, Fukuoka, and Kure, as well as agricultural villages in the nine prefectures that surrounded these cities. In addition, coal mines in Fukuoka Prefecture were included in the second survey of the above mentioned areas. The continuation of these surveys, the third and the fourth, were conducted in August (summer) and November (autumn) of 1946 to study the link with harvest or season. The results of these four surveys elucidated, for the first time, the Japanese people’s changing dietary habits over the time periods in question as well as the degree and the course of nutritional disorders in their entirety. These nationwide nutrition surveys commissioned by the government (Ministry of Health and Welfare) can be called epidemiologic surveys. The author would like to attempt an overview of the surveys based on several reports^29^^-^^36^ related to the surveys. However, the author must admit that he by no means could obtain all the relevant reports.

## THE POSTWAR PUBLIC’S NUTRITIONAL INTAKE AND NUTRITION DISORDER: FREQUENCY OF MALNUTRITION DYSTROPHIA

### Nationwide nutrition survey of 1945

Despite the postwar reorganization of the Japanese government’s departments and bureaus, the Nutrition Section of the Ministry of Health and Welfare remained intact. During the war, the section’s staff had engaged in nutrition surveys of residents. The ministry’s Nutrition Section also played a leading role in planning and conducting this survey.

In cities with populations of over one million, 1% of the residents were sampled, in cities with populations of 500,000 to one million, 2% were sampled, while in rural areas that neighboring these cities, the same number were sampled as in the cities themselves. The sampled residents’ physical statuses were checked. Further, in half the groups dietary surveys were conducted. Thus these surveys followed excellent epidemiologic survey methodology.

According to Heigo Kuwabara,^32^ in charge of the Tokyo Nutrition Survey, the surveyors formed their plan over repeated staff meetings: deciding on method, creating necessary forms, choosing sites for medical examinations, organizing medical teams, drawing up lists of the demographic composition of the survey areas, preparing subject lists, presenting explanations at area liaison meetings, and requesting community cooperation. The surveys included medical examinations via interview and physical status examination. Physical status was examined by teams of seven persons including physician, nurse, public health nurses, and nutritionist. Following a predetermined procedural outline, the group conducted medical examination by interview, physical status examination, and interviewed on dietary habit, then, calculated of nutrition values from interview slips and tallied results. The number of persons and households to be sampled was proportional to the population of individual wards in Tokyo or rural villages. In selecting persons to be surveyed, consideration was paid to avoid sample bias due to sex, age, occupation, or economic condition. Railroad workers were included as part of the surveyed. Over the four surveys, as much as possible the same person would carry it out on the same household. Physical examinations were held at prescribed places. Participants were asked to follow instructions in the survey form. In set form, the quantity of food intake was to be entered in detail for three consecutive days; then, a nutritionist would check the entries, evaluate them, and tallied results. Nutrition disorder-related items checked in physical examinations were: height, weight, anemia, stomatitis, cheilitis, glossitis, patelloadductor reflex, edema, keratosis pilaris, chronic diarrhea, menstruation delay or amenorrhea, bradycardia, insufficient breast milk secretion, corneal xerosis (keratomalacia), and hypo-osteosis. Subjective symptoms of malnutrition dystrophia included: weariness, fatigue, frigid hand and foot, skin change, keratoderma (hair root), edema (face: later limbs), bradycardia, reduced breathing, low body temperature, low blood pressure, excessive passage of urine, angular cheilitis, glossitis, chronic diarrhea, psychoneurotic symptoms (somnipathy, headache, dizziness, tinnitus, hypomnesia, etc.) The symptoms listed above were based on research work that reported these as related to malnutrition dystrophia. Another report by Hibi ^33^^)^ gave detailed account of the physical examinations conducted in Ushigome ward, Tokyo. Thus the surveys appear to have been a large scale national project in the context of the time. Their reports give the impression of an excellent epidemiologic survey of area residents. The accuracy of the data was judged to be good. While the survey was conducted at the difficult time that followed the war’s end, its success was due to the research participants’ high competence. Other factors that contributed to success were that the survey’s contents were deeply felt by the survey workers themselves as well as willing cooperation on the part of those surveyed.

According to Kuwabara, the first survey in Tokyo involved 31,968 ward residents and 3,514 households. The average energy intake of ward residents was 1971.5kcal with 64.2g protein (13.6g animal-origin) per day. Only 54.7% of energy came from rationed food, the supplementary food was purchased unofficially (on the black market.) Only a very small proportion of the energy came from home gardens or other sources. The above statistics told that surplus food from farmers in surrounding areas had to some extent entered the black market.

When classed by wards, energy intake in Tokyo differed depending on area, in a range of 1670.6-2953.2kcal. Of 3,393 persons surveyed by occupational grouping, wage-earners’ energy intake was 1104.5kcal, 2288.2kcal for merchants, 2150.4kcal for laborers, 2261.1kcal for factory workers, and 2462.5kcal for those unemployed; the lowest figure was for wage-earners. Reasons offered for the majority of ward residents’ ability to purchase food on the black market included people’s eagerness to purchase food; some had ready cash received as severance pay following loss of employment, drop of monetary value with inflation due to material shortages or rumors of devaluation and conversion to newly printed money.^22^

The poor crop of the autumn of 1945 made difficult the planning of food supplies for the coming winter. The survey in the off-crop month of May next year found that the total energy intake of Tokyo’s ward residents had dropped to 1351.9kcal. However, GHQ’s emergency food aid fortunately began in April in response to the surveys of the actual condition of food supplies. This action by the GHQ resulted in an August 1946 increase of an average energy intake of 1827.8 kcal -- despite a previous estimate that food supplies for that month would be at their lowest. On the other hand, for the worst month of May total average energy intake of farmers in Tokyo was 2075.5kcal, and 2079.6kcal in August. While low as it was, Tokyo farmers’ total energy intake was always higher than that of urban residents. Findings of the survey of November 1946 included: total energy intake of Tokyo ward residents increased to 2051.4kcal; farmers’ energy intake could go back to 2366.6kcal. The Railroad Bureau was chosen to participate in this survey, because it had always been cooperative with various surveys and known as a group that had contributed accurate data. In the survey of May 1946, total energy intake of railroad workers was a low 1685.8kcal suggesting that few purchases were made from external sources. It might be due to their attitude of mind as government employee. While railroad workers’ total energy intake increased, 1896.3kcal in August and 2002.4kcal in November, these numbers were lower than those of other groups’. These railroad-worker households provided an important example of the difficulties that wage earners faced in supplementing their food rations.

The quantity of protein intake in November 1946 in Tokyo lay in the vicinity of 55g a day. Intake of animal-origin protein by ward residents was 14.5g, and 2.7g for farmers. This suggested that while high in energy intake, the quality of the farmers’ diet was poor, mostly centering on cereals. Surveys^34^^-^^35^ described diet habit and nutrition condition in very vivid detail as well as their relationship to household budget over small areas within Tokyo. Here again, are proofs that by the end of 1946 diet had improved considerably.

### Prevalence of malnutrition dystrophia among Tokyo ward residents

Below are malnutrition dystrophia-related prevalences according to the 1946 survey.

As of May 1946, prevalence of malnutrition dystrophia among Tokyo ward residents as a whole was 36.4%, 21.0% for farmers, and 49.1% for railroad workers -- an extremely high frequency. Prevalence was high in the group of 30 years of age and older; no age differences were observed among railroad workers, and every age group displayed marked nutrition disorder. Breakdown by symptoms were: a high rate of 69.5% for anemia, approximately 3% for keratosis pilaris, glossitis and stomatitis, 2.3% for chronic diarrhea, 7.6% for angular cheilitis, 7.2% for bradycardia, 4.0% for edema, 12.2% for disappearance of the tendon reflex, and 8.6% for menstruation disorder. By November 1946, prevalence rates for the above symptoms were: 4.0%, 1.0%, 2.0%, 0.8%, 6.0%, 1.4%, 1.8%, 9.1%, and 16.5%, respectively -- showing a little improvement, but still rather high. Reduction in body weight for urban residents in February 1945 was 55% and 34% for farmers: by November 1946, both groups’ weight recovered to 18.4% and 14.3%. However, weight loss for railroad workers was a high 30%. Not all the persons with the above symptoms suffered from malnutrition dystrophia, rather, they might be termed a reserve army for malnutrition dystrophia. Omori’s comprehensive conclusion over the results was that an estimate of 5% in Tokyo suffered from malnutrition dystrophia -- indeed a great number of patients. While the survey results from other cities were similar, as was stated previously there were considerable regional differences.

### Results of the 1947 Nationwide Nutrition Survey

In February, May, August, and November 1947, four expanded surveys were conducted. Survey areas included nine cities (Sapporo, Sendai, Tokyo, Kanazawa, Nagoya, Osaka, Kure, Matsuyama, and Fukuoka) and 27 prefectures (Hokkaido, Iwate, Miyagi, Ibaraki, Tochigi, Gunma, Saitama, Chiba, Tokyo, Kanagawa, Shizuoka, Aichi, Toyama, Ishikawa, Fukui, Shiga, Kyoto, Osaka, Hyogo, Wakayama, Okayama, Hiroshima, Ehime, Kochi, Fukuoka, Saga, and Kumamoto), and the Railroad Bureau (Tokyo). The same survey methods were applied.

Results from these surveys indicate that in general total energy intake increased, ranging between 1736-1977kcal per day in urban areas and between 2012-2178kcal in farming villages. Protein intake also increased from 58g to 63g. While total energy intake differed little in urban or farming areas, animal-origin protein intake in urban areas ranged 14-17g, but it was 6-8g in farming areas. Looking at inorganic acids, calcium deficiency was notable in both areas. Also noted was vitamin A and B_2_ deficiency. In agricultural villages, vitamin B_1_ intake was also inadequate. Looking at foodstuff types, cereals and potatoes (potato, sweet potato, taro and other starchy roots) were more frequently served in agricultural areas than in urban areas; fish and sea weeds were eaten less frequently on farms. A low consumption of yellow and green vegetables in agricultural areas was a surprise.

The prevalence of nutrition disorders showed a decrease when comparing the FY 1947 with the 1946 surveys. Yet, the occurrences of menstrual disorder, disappearance of the tendon reflex, angular cheilitis, and anemia were not negligible. More often observed in agricultural areas were glossitis, angular cheilitis, and bradycardia. Problems with breast milk insufficiency were common. Many cases of infant nutrition disorders were also found.

In summary, 1945 was a year of poor crops worldwide. Preferential food aid to a defeated Japan was a problem. Nevertheless, the helping measures taken by GHQ upon their recognition of the results of fact-finding surveys and of the nutritional condition of Japan’s residents’ made it possible to weather the worst of the periods of starvation: Among these measures were the emergency food imports of February-April of 1946, the release of food formerly reserved by the Japanese army for their own use and rice imports following April 1946. A so-so rice crop as well as the bountiful harvest of sweet potatoes of 1946 were life savers. The issuance of an ordinance ensured delivery of rice. Social disparities did emerge as a result of so-called black market purchasing power which affected the frequency of nutrition disturbances.

The following are findings from Arimoto’s collective report^34^ on the eight surveys conducted between February 1946 and November 1947. In urban areas, energy intake increased from 1500-1700kcal to 1880-2000kcal with few differences as to kinds of nutritional elements. In agricultural areas energy intake lay in a range of 2000-2200kcal. However, there, protein and fat contributed less to total energy intake than that in urban areas. Intakes of vitamin A, B_1_, and B_2_ were generally low. In urban areas, 56-67% of energy came from rationed food; the rest depended on individual sources. Many there experienced weight loss. In these urban areas, anemia and edema were common; breast milk secretion also showed decrease. More cases of angular cheilitis, glossitis, disappearance of the tendon reflex, or bradycardia, were reported in agricultural areas than in urban ones. A high prevalence of specific nutritional disorders was reported in agricultural areas despite a generally higher energy intake. This appears to have been a reflection of low intakes of specific nutritional elements. As previously stated, low income homes, e.g. those of war victims or of the jobless, found it difficult to purchase food to supplement rations. The nutritional condition of the homeless (including homeless children) or institutional inmates was quite poor, with high prevalence of malnutrition dystrophia. In November 1946, considerable quantities of aid material, e.g. food, clothing medicines, etc., sent by LARA (Licensed Agencies for Relief in Asia), a private organization, arrived. LARA aid continued until it was discontinued by 1952. Distributed exclusively to institutional inmates, LARA aid greatly helped these persons. During those times too, new social disparities, created by differences in income earnings, occupation, family composition, or the like added to what we have called the size of the “reserve army” for malnutrition dystrophia.

The FY 1948 Survey was conducted in the same manner as those previously described. The report of some improvement emphasized the importance of food production, its processing, and nutrition guidance. While malnutrition dystrophia should rapidly decrease with improving food supply, the decrease of deaths caused by malnutrition dystrophia was moderate due to the presence of intervening factors other than food.

## POSTWAR MORTALITY CAUSED BY MALNUTRITION DYSTROPHIA

The author has not found any truly nationwide epidemiologic survey of malnutrition dystrophia. In 1947 when dietary habits in Japan began to improve, publication of the vital statistics in Japan resumed. The author attempted to trace the mortality trends for malnutrition dystrophia from numbers of deaths recorded in the vital statistics tables.^37^ Owing to some diagnostic errors generally contained in such mortality statistics, their accuracy may be a problem. With common ailments, the rate of diagnostic errors based on comparison between medical certificate and postmortem examination is thought to be approximately 10-15%. There may also be overlooked cases. However, the author believes that approximate trends can be estimated from Japanese mortality statistics sources.

The number of deaths due to malnutrition dystrophia was 7,476 in 1947, 3,474 in 1948, 2,723 in 1949, 4,680 in 1950, 2,299 in 1951, 1,767 in 1952, 1,782 in 1953, 1,491 in 1954, 1,177 in 1955, 1,166 in 1956, 1,130 in 1957, 947 in 1958, 886 in 1959, and 754 in 1960. By three years later in 1951, the number had decreased by half. Ten years later, the number had gone down to below 1,000. By 1965, the number was below 500. It is noteworthy that the number had remained over 5,000 until around 1950 -- once elevated malnutrition dystrophia mortality did not diminish until after a considerable passage of time.

According to Inoue’s report,^9^ the number of malnutrition dystrophia patients was at a maximum in 1945. By 1946, the number had decreased by 60%, and it was lower yet in 1947. Based on statistics from a particular facility, in the year of the war’s end in 1945, the estimated number of malnutrition dystrophia deaths may have been at least 15,000. The reasons for the 1950 temporary increase of deaths were not clear. The author presumes that the great migration from wartime places of evacuation to metropolitan areas in search of work was a relevant factor. At that time of housing and food shortages, such moves must have been mentally as well as physically quite stressful. The fact of an increase of general mortality at the time backs up the author’s assumption. However, in the following year, the all-cause mortality rate would also decrease.

According to Omori,^8^ the prevalence of malnutrition dystrophia in 1947 lay in the vicinity of 5%. Supposing the over age 20 year population at the time to be 40 million, the estimated number of malnutrition dystrophia patients was 2 million. If the assumption that 5% of them were severe case is correct, the total number of sever patients’ number would be 100,000. Assuming 1945 deaths from nutrition dystrophia numbered 15,000, fatality rate would be as high as 15%. After 1947, incidence, prevalence, and mortality of malnutrition dystrophia may be presumed to have fallen. However, the approximately 3,500 deaths in 1948 suggested that the malnutrition dystrophia patients were still numerous.

The period of a sufficient total energy intake coupled, however, with an insufficient intake of necessary nutritional elements lasted over 10 years. Improvements in other living as well as social conditions were also slow to come. In this environment, over 1,000 annual deaths from nutritional dystrophia continued to be recorded until 1958. The author feels that it was natural for government officials to regard continued annual national nutrition surveys in Japan to remain a necessity in order to observe current status.

Despite the continuing post -1955 more than 1,000 deaths due to malnutrition dystrophia, survey reports on the illness by region or group have become rare since that year. In the author’s memory, too, interest in nutrition disturbances has quickly faded and has been replaced by concerns with other problems, such as tuberculosis. In the author’s opinion, pathogenic mechanism or characteristics of malnutrition dystrophia, an illness that claimed so many victims, could have been further pursued even in the abnormal times of end-of-war Japan. However, during the times considered, neither the public nor the private sectors had the luxury of focusing on said questions. The author regrets that research on malnutrition dystrophia has not been continued. Important questions relevant to the illness have not been addressed, including how dietary imbalances may contribute to the illness, how other external factors may be involved, how the patient’s constitution may be a relevant factor, how said dystrophia may differ from other nutrition-related diseases such as beriberi or diabetes, etc., what relationship it may have to general metabolism, endocrine secretion, brain or neurological functions, or the like. If pursued, findings from research on topics such as listed above would have even been useful to studies of our contemporary lifestyle diseases due to the present habits of excessive eating.

## CONCLUSIONS

The author has thought his writing a short history of malnutrition dystrophia had been a fantasy. However, he feels to have been lucky to have been able to do this overview of malnutrition dystrophia with the aid of the unexpected presence of many records on the topic. Research on the following issues has proved important: the pathogenic mechanism of malnutrition dystrophia, especially external factors that facilitate its onset, the illness’ complications, the role of internal factors, condition’s relationship to beriberi or to other metabolic diseases, as well as the clinical study of relevant mental disorders. In addition the author hopes for further finding of literature sources presently unknown to him.

Malnutrition dystrophia can be caused not only by nutrition deficiency, but also by nutrient excess. Comparative research, e.g. how malnutrition dystrophia fundamentally differs from present obesity or diabetes, or different ways metabolism, internal secretion, or nervous system may relate to it, may yield fruitful results.

Lastly, the national nutrition surveys, major undertakings immediately after the war’s end had been shown to have great value. As previously described, nutritionists trained in Japan who were active in these modern surveys which ended in great success have been found worthy of having their names recorded. In April 1947 there took place the reopening of the National Nutrition Research Institute with increasingly rich assets: authorized professional staff positions, further substantiation of national nutrition surveys, and the accumulation of materials that increase every year. While the term “epidemiology” does not appear in the name, the “National Nutrition Survey of Japan” is basically an epidemiologic survey that forms the basis of health or disease prevention research, or of countermeasures. Meanwhile, the rekindled flare- up of beriberi, once a ravaging yet now an easily preventable disease, is a problem for the future that must be fully examined.

## Attachment: Death Due To Beriberi

Research by Inoue and Omori has defined beriberi to be a different disease from malnutrition dystrophia. Because the mortality of beriberi increased together with that of malnutrition dystrophia, the situation is briefly described below.

Beriberi is also a nutritional disorder, the cause of which is identified as vitamin B_1_ deficiency. Additionally, lifestyle factors greatly contribute to increase beriberi incidence. As is well known now, overeating of polished rice was the major cause for high beriberi mortality during the Meiji and Taisho era. Owing to collective campaigns against polished rice by the public and private sectors as well as dietary nutrition education regarding vitamin B_1_, for a time during the 1930s the number of beriberi patients drastically decreased. However, during World War II the number of beriberi cases apparently increased again. After that war, significant increases in beriberi deaths were also noticed.

According to Inoue’s statistics on patient numbers,^9^ 1946 morbidity for vitamin deficiency, centering on beriberi, was twice as high as that for malnutrition dystrophia. As of The Health and Welfare Ministry’s national nutrition survey of May 1946 found, the percentage of patients with symptoms that fit beriberi symptoms was 11% in urban areas and 7.2% in agricultural areas (as of August): prevalence was still high in August 1948, 8.6% in urban areas and 8.7% in agricultural areas. In May 1948 survey, prevalence among coal mine workers was 12% and among railroad workers, 12-18%. Namely, beriberi cases increased nationwide numbering by over several million.

Beriberi deaths from the vital statistics in Japan^37^ were as follows: 8,596 in 1947, 6,281 in 1948, 5,562 in 1949, 8,968 in 1950, 3,202 in 1951, 2,413 in 1952, 1,908 in 1953, 1,533 in 1954, 1,126 in 1955, 957 in 1956, 746 in 1957, and 580 in 1958. Far more beriberi deaths occurred than those from malnutrition dystrophia; however the former finally came down below 1,000 in 1956.

The nationwide estimate for patients with beriberi at one time could have been over 10 million. Yanagi^38^ ascribes the reason for the high prevalence to the loss of effectiveness of an ordinance that prohibited eating polished rice instead encouraging rice with germ. As the ordinance naturally became obsolete with the war’s end, people, forgetting the horror of beriberi, once again began to polish rice. With the rice supply improving, polished rice diet become popular. The majority of households in Japan used to polish rice in a 2 liter bottle. Insufficient intake of protein, fat, and other nutritional elements such as vitamins of the time, polished rice eating resulted in increased beriberi patients. It was ironical that, either during or after the war, prevalence of beriberi was higher among laborers who received higher rice rations than the general public -- they ate polished rice. In 1947, beriberi mortality was highest in Nara and Nagano prefectures; also high was in rice-producing Shikoku, Kyushu, Kansai, and Tohoku regions. Relatively high beriberi mortality among infants was associated with breast milk of mothers who suffered from beriberi, a big problem in the field of maternal and child health. Another contributing factor to high infantile mortality was below average vitamin B_1_ intake among mothers, even if they were not on polished rice diet. Even while survey-related professionals involved had been instructing the importance of vitamin B_1_ intake, the generation ignorant of the horror of beriberi did not follow instruction, though they might have understood it in their minds. The author thinks the root of the problem lay in weakened confidence in a government that had led the public into war or in local administrators in general, or in anti-authority defiance, indifference to diet or health, or attitude toward life. With the outbreaks of “Give Us Rice” movements, demand for food in quantity, rather than interest in health education, became prominent. In the background of this situation lay an inability to change the emphasis on quantity of food supply and delays in reversing earlier health guidance measures. Later on, the factors described above greatly affected overall dealing with chronic diseases.

The facts described above bespeak the lesson that without continual hygiene education, a high incidence of a disease or an epidemic may occur even after diseases’ pathogenesis is clarified or practical preventive measures are identified. The public tend to forget once mortality has become low: when the threat of fear slips away, disease countermeasures will be relaxed. As a result, the public will once again have to make identical sacrifices, exert labor, and pay expenditure. In Japan where we live on rice, we must engrave in our minds that beriberi is an old yet always a new problem.
